# Using bioinformatic and phylogenetic approaches to classify transposable elements and understand their complex evolutionary histories

**DOI:** 10.1186/s13100-017-0103-2

**Published:** 2017-12-06

**Authors:** Irina R. Arkhipova

**Affiliations:** Josephine Bay Paul Center for Comparative Molecular Biology and Evolution, Marine Biological Laboratory, Woods Hole, MA 02543 USA

**Keywords:** Mobile genetic elements, Classification, Phylogeny, Reverse transcriptase, Transposase

## Abstract

In recent years, much attention has been paid to comparative genomic studies of transposable elements (TEs) and the ensuing problems of their identification, classification, and annotation. Different approaches and diverse automated pipelines are being used to catalogue and categorize mobile genetic elements in the ever-increasing number of prokaryotic and eukaryotic genomes, with little or no connectivity between different domains of life. Here, an overview of the current picture of TE classification and evolutionary relationships is presented, updating the diversity of TE types uncovered in sequenced genomes. A tripartite TE classification scheme is proposed to account for their replicative, integrative, and structural components, and the need to expand in vitro and in vivo studies of their structural and biological properties is emphasized. Bioinformatic studies have now become front and center of novel TE discovery, and experimental pursuits of these discoveries hold great promise for both basic and applied science.

## Background

Mobile genetic elements (MGEs), or transposable elements (TEs), are discrete DNA units which can occupy varying positions in genomic DNA using the element-encoded enzymatic machinery [1]. The further we advance into the era of extended genomics, which now includes personalized, ecological, environmental, conservation, biodiversity, and life-on-earth-and-elsewhere genomics and metagenomics, the more important it becomes to fully understand the major constituents of genetic material that determines the blueprint of the living cell. It is now common knowledge that, in eukaryotic genomes, sequences corresponding to protein-coding genes often comprise only a few per cent of the genome. The bulk of the poorly understood genetic material, labeled “dark matter” by some researchers and “junk DNA” by the others, consists mainly of TEs and their decayed remnants, or represents a by-product of TE activity at critical time points in evolution.

The advent of next-generation sequencing technologies led to an unprecedented expansion of genome sequencing data, which are being generated both by large consortia and by small individual labs, and are made widely available for data mining through publicly accessible databases. Due to their high proliferative capacity, TEs constitute a substantial fraction of many eukaryotic genomes, making up to more than one-half of the human genome and up to 85% of some plant genomes [2]. The necessity to sort out these enormous amounts of sequence data has spurred the development of automated TE discovery and annotation pipelines, which are based on diverse approaches and can detect known TE types in the newly sequenced genomes with varying degrees of success (reviewed in [3, 4]).

In this review, some of these methods and their applicability to different types of TEs are evaluated from the user’s perspective, aiming to provide a brief overview of the historical and current literature, to assist the prospective genome data-miners in the choice of methodologies, to provide an updated picture of complex evolutionary relationships in the TE world, and to encourage the development of new bioinformatic approaches and tools aimed at keeping up with the ever-changing nature of the currently accepted TE definitions. It is intended to stimulate further discussions in the TE community regarding the importance of more uniform and standardized approaches to TE identification, classification, and annotation across species; to underscore the dominance of in silico studies as the current forefront of TE discovery; and to emphasize the utmost importance of in vitro and in vivo studies of TE biology in the ultimate quest for understanding the rules of life.

### TE identification: Principles, tools, and problems

The variety of TE detection tools in newly sequenced genomes makes it unpractical to compile a full list of such tools (for a few recent lists, see [5, 6]). Nevertheless, it would be fair to say that no single tool can be applied universally across all species for all TE types: tools that detect repetitive sequences in genome assemblies de novo in all-by-all comparisons can generate a repeat library that would only partially overlap with a k-mer based repeat library, or with a homology-based library. Thus, comprehensive software packages that can integrate information from a combination of several TE detection tools into a composite library, such as TEdenovo (Grouper, Recon, Piler, LTRharvest) in the REPET package [7] and RepeatModeler (Recon, RepeatScout, TRF) (http://www.repeatmasker.org), are currently dominating the field of TE identification. Other tools can search for over-represented repeats in unassembled sequence reads, employing k-mer counts, machine learning, and low-coverage assemblies ([8–10] and references therein). By a practical operational definition, most programs divide TEs into families by the 80–80-80 rule: nucleotide sequence identity between members of the same family longer than 80 bp is 80% or higher over 80% of its length [11]. While in some organisms this approach may create an unnecessarily high diversity of families, and a 75% identity threshold, often well-supported by phylogeny, could also work well, it would probably be unpractical to introduce major changes at this point.

While the above packages represent a good starting point, some of the associated problems, such as the ***mutual dependence of repeat identification quality and repeat library composition***, have been discussed in [6], and, from our experience, the list of problems can be easily expanded. Construction of a comprehensive TE library remains the most critical point for their subsequent annotation and analysis. However, even the integrated tools for TE detection in eukaryotic genomes are not interchangeable, as they were initially targeted towards specific taxonomic groups such as plants or mammals, which share some of the repeat types but not the others. For instance, the ***structure-based identification*** component in TEdenovo categorizes any two repeats separated by a spacer as LARDs or TRIMs (non-autonomous LTR retrotransposons abundant in many plant genomes) [12, 13]. However, these TE types are not too prominent in animal genomes: we found that, when applied to bdelloid rotifers, this tool retrieves mostly segmental duplications unrelated to TEs [14].

These microscopic freshwater invertebrates also highlighted several other organism-specific problems in TE annotation, such as the ***over-abundance of very low-copy-number TEs*** (1–2 copies per genome), which are not being recognized as repeats in the first place; and ***degenerate tetraploidy***, which lowers the sensitivity even further, due to the need to increase the minimum copy number threshold for repeat detection from 3 to 5 to avoid inclusion of host gene quartets. In bdelloid genomes, one-quarter of TE families went undetected by the TEdenovo and ReAS [15] tools, and could be identified only during manual curation [14]. On top of all that, bdelloids contain a ***previously unknown type of giant retroelements*** with multiple ORFs not associated with known TEs, which also escaped automated recognition [16].

Finally, among the downsides of an all-inclusive de novo repeat library is the almost inevitable incorporation of ***host multigene families***, if these are composed of members with sufficient sequence similarity. While REPET developers did address this problem in one of the releases, the solution was based on supplying a host gene set. However, unless a closely related reference genome with a thoroughly curated gene set is available, such gene set in the first approximation will inevitably contain at least some TE sequences, thereby excluding them from the “cleaned-up” library and creating a circular problem. Thus, the presence of host genes may be an inevitable trade-off in a fully automated repeat library free of manual curation. In rotifers, such genes turned out to be the biggest contributors to the “unknown” TE categories, constituting at least one-half of the TEdenovo library, and can substantially inflate the TE content if left unaccounted for.

In sum, while TE identification tools have improved dramatically since the early days of comparative genomics, and novel methods are constantly being developed, it is important not to lose sight of biological properties of TEs and their hosts, and to make every effort to inspect, at least partially, the outputs of even the most widely used computational pipelines, before drawing any far-reaching conclusions in unfamiliar genomes. Furthermore, the variety of tools makes it difficult to compare published information across diverse genomes, which most likely have been measured with different yardsticks. Thus, for the most critical comparisons, a set of genomes should be processed with the same toolkit to achieve meaningful results.

### TE classification

An unclassified TE library is of limited use until it is subjected to classification. Once the repeat libraries are generated, they are run through a classification pipeline which can assign automatically numbered repeats to known categories. Since TEs are polyphyletic, i.e. do not share common ancestry, a brief overview of the current TE classification systems would be appropriate for understanding how different TE groups relate to each other.

TE classification has long been, and continues to be, a subject of debate [11, 17–19], although certain standards had to be established to address the urgent needs of comparative genomics. The main approaches to TE categorization, which rely on different criteria, are described below.

#### RNA or DNA-based?

The earliest classification scheme by Finnegan in 1989 [20] introduced an important dichotomy, i.e. whether the TE employs RNA as a transposition intermediate for its mobility (Class I, or retrotransposons) or does not (Class II, or DNA transposons). This principal subdivision of TEs into two major types traditionally relies on the nature of their transposition machinery: Class I elements code for a reverse transcriptase (RT), which utilizes an RNA intermediate in the transposition cycle; and Class II elements code for a transposase (TPase), which does not employ any RNA intermediates and operates entirely at the DNA level. While the diversity of known TEs has increased dramatically since 1989, the role of RNA in the transposition cycle remains one of the most useful practical criteria in guiding the initial TE classification. A homology-based search can easily determine whether a given TE family codes for an RT, or, using a more simplistic terminology, represents a “copy-and-paste” TE. If it does not, it can be classified as a DNA TE, and would then fall into one of three broad subclasses: “classical” DNA TEs coding for a DDE TPase, most of which are referred to as “cut-and-paste”; rolling-circle, or “peel-and-paste” replicative TEs coding for a replication initiator-like protein (Rep/HuH); and “self-synthesizing” DNA TEs coding for a protein-primed B-type DNA polymerase [21–23]. Thus, while all RTs share a common catalytic core with the so-called “right-hand” fold, the term “transposase” designates several unrelated groups of TEs, unified only by the lack of an RNA intermediate in their remarkably diverse transposition cycles.

#### Mechanistic approaches

Studies of the molecular mechanisms of transposition and high-resolution 3-D structures of TPase complexes led to designation of five major TE groups in accordance with insertion mechanisms and the corresponding enzymes responsible for integration, as outlined by Curcio and Derbyshire [21]: RT/En; DDE TPases; Y-TPases (tyrosine); S-TPases (serine); and Y2-TPases (rolling-circle). The DDE, Y, and S TPases perform “cut and paste” transposition, while RT/En and another DDE subset perform “copy and paste”, with further subdivisions for the first (“out”) and second (“in”) steps (cut-out, paste-in; copy-out, copy-in; etc.) and formation of a hairpin intermediate during excision. This classification applies to both prokaryotic and eukaryotic TEs, and therefore provides a unified picture of interactions between TEs and host DNA required for mobility. However, the focus on integration mechanisms leaves out the replicative component, which may pose a practical difficulty in classifying the vast majority of eukaryotic retroelements.

Hickman et al. [24, 25] focused on the same four types of transposases, as specified by the chemistry of the transposition reaction - DDE, Tyr, Ser, and Y1/Y2 (aka HuH), and have enriched the mechanistic aspects of this classification by placing additional emphasis on 3-D structural features of enzymes performing these diverse biochemical reactions. Overall, the mechanistic approach should be applauded for bringing together prokaryotic and eukaryotic TEs, however it presents a somewhat simplified view of retrotransposition, which is centered on integration, while in fact it involves a rather complex sequence of diverse events.

For retrotransposons, prokaryotic and eukaryotic, Beauregard et al. [26] proposed to divide them into extrachromosomally-primed (EP) and target-primed (TP), in agreement with their priming mechanism. According to this principle, most retrotransposons, including group II introns (G2I), would fall into the TP category, with EP having emerged much later, in the course of evolution of retrovirus-like elements. However, assigning a specific priming mechanism to the poorly studied TE types may be challenging until it is confirmed experimentally.

#### Homology-based approaches

At present, the most common approach to identifying TEs in genomic sequences is by homology to known enzymatic activities that are already known to be associated with mobility of a certain TE type, which in turn can be tied to a specific mechanism of transposition. Although this approach may result in misclassifying domesticated TE-derived proteins as TEs, in most cases a DNA segment coding for an RT or TPase can be safely classified as a TE. While the non-enzymatic components, such as *gag* genes, also belong to the set of TE hallmark genes, they exhibit much less conservation due to the lack of catalytic residues, and are therefore more difficult to recognize than their enzymatically active partners, which usually serve as an “ID card” for any autonomous TE. Thus, the molecular signature of a TE-encoded protein with an enzymatic activity routinely guides its molecular systematics.

#### Eukaryotic TEs: Current classification

The Wicker and Repbase TE classification systems [11, 17] were designed to target eukaryotic TEs, and addressed the practical needs in eukaryotic comparative genomics by providing a streamlined hierarchical approach to sorting through TE content in gigabases of genomic DNA. In Wicker et al. [11], the “order” category was borrowed from taxonomy to fill in the gap between “class” (I or II) and “superfamily”, although “subclass” is still widely used for designation of the same category. Orders (with numbers of superfamilies in parentheses) include LTR (5), DIRS (3), PLE (1), LINE (5) and SINE (3) for class I; and TIR (9), Crypton (1), Helitron (1), and Maverick/Polinton (1) for class II. Each TE family is assigned a three-letter code based on its class, order, and superfamily, with the first letter being R or D for retrotransposons and DNA transposons, respectively (as in RIL for RNA/LINE/L1). This three-letter code was implemented in the REPET package [7]. In practice, however, identification rarely proceeds all the way to the superfamily level, especially when applied to understudied taxa, and mostly results in ambiguous designations such as RLX or RXX. Additionally, as mentioned above, it can easily mis-annotate non-autonomous TEs, which can only be recognized by their structural features (e.g. TRIM and LARD [27]), assigning essentially any pair of repeats separated by a spacer to these non-autonomous LTR retrotransposons, without taking into account conserved terminal nucleotides or target-site duplications (TSD). The Repbase classification system, which is more heavily focused on animals, provides the resource for homology-based RepeatMasker annotation, which has a built-in classification tool, and employs four major subclasses (DNA, LTR, ERV, non-LTR), with further subdivisions into superfamilies. The RepClass classification tool employs four subclasses (DNA, LTR, non-LTR, Helitron), and identifies class (C), subclass (SC), and superfamily (SF), accounting for homology, structural features, and TSDs [28].

#### Prokaryotic TEs: Should different domains of life be integrated?

Bacterial and archaeal mobilomes share a lot in common with eukaryotic mobilomes in mechanistic terms, but they nevertheless exist in parallel universes. The ISFinder database [29] contains insertion sequences (IS), which code for DNA transposases classified in 26 families, and may or may not carry accessory or passenger genes. It serves the bacterial community since 2006, and provides the ISsaga pipeline [30] that facilitates IS identification and semi-automatic annotation in sequenced bacterial genomes. Separate databases exist for group I introns [31] and inteins (also called protein introns) [32], which use specialized endonucleases for their integration. The group II intron database [33], which offers its own identification and collection pipeline [34], is the resource for bacterial retroelements. Homing endonucleases (HEN) can be associated with both group I and group II introns, as well as inteins; out of six known types (HNH, His-Cys box, LAGLIDADG, Vsr (EDxHD), PD(D/E)XK, and GIY-YIG) [35], at least two can also be found in eukaryotic TEs (GIY-YIG, as part of PLEs, and PD(D/E)XK or REL, as part of non-LTR TEs) [36, 37]. Serine TPases (IS607-like) might possess eukaryotic homologs [38]. Finally, the rolling-circle replication (RCR) IS200/IS605 TE families (also termed “peel-and-paste”, or Y1 [23]), which utilize a single-stranded DNA intermediate, can be loosely paired with eukaryotic Helitrons (Y2), for which an RCR model of transposition has been proposed and circular intermediates detected [39, 40].

An argument for integrating TE systematics across domains was put forward by Piégu et al., who provided an overview and evaluation of the existing TE classification systems, aiming to merge similar TE groups from different domains of Life [19]. They argued that, despite the substantial degree of similarity between prokaryotic and eukaryotic TEs, their classification systems remain disconnected, and pointed out the need for a universal classification system that would embrace all kingdoms of life. They also argued that TE inventories should include the “overlooked” elements such as self-splicing introns, inteins, and even spliceosomal introns. In a sense, spliceosomal introns can be regarded as non-autonomous elements which rely on the *trans*-acting spliceosomal machinery for excision from RNA, and share a common origin with retroelements through one of its principal components, Prp8, the core of which was derived from an RT through the loss of catalytic residues [41, 42]. Nevertheless, even if introns originated from mobile elements, there are conflicting views on the mode of their dispersal: competing with the reverse-splicing model is the view that spliceosomal introns take their origin from non-autonomous DNA transposons [43]. Overall, the recommendation to focus attention of the TE research community on taxonomy issues through a gradual process of collegial discussion in the frameworks of an international society [6] merits consideration and support.

### TE classification in the context of phylogeny

It has been argued that a viable TE classification system should reflect their phylogeny [18], although the polyphyletic nature of TEs would not make this task easy [44]. The genomes of host species contain large numbers of co-evolving genes, which can be used to infer relationships between these species using multi-gene analysis, based either on superposition of many individual gene trees, or on building species trees from concatenated sets of conserved core reference genes. In contrast, phylogenetic studies of TEs do not have the luxury of utilizing multigene sets. On the contrary, even a single ORF could be composed of multiple domains with different evolutionary histories and different degrees of conservation (see below). Thus, determining whether any specific groups of mobile elements are more closely related to each other than to other known groups is a much more daunting task than determining phylogenetic relationships between their hosts, since it usually boils down to one-gene phylogenies. The relative structural simplicity of most TEs often prevents researchers from determining whether some of them are more closely related to the presumptive ancestral forms than the others, due to insufficient phylogenetic signal.

#### Conventional phylogenetic analysis

Phylogenetic methods have been used to infer the evolutionary history of TEs since the emergence of such methods in mid-80’s. In the early days of TE analysis and molecular phylogenetics, when nucleotide sequences were still being printed on journal pages and parsimony methods ruled the field, nucleotide sequences of Alu and L1 retroelements were already revealing their peculiar subfamily structure and the unusual pattern of succession of master copies [45–47]. Indeed, mammalian genomes create a perfect setting for inferring phylogenetic histories of TEs in parallel with their hosts, due to their convenient biological property of accumulating large amounts of “junk DNA” as a “fossil record”, instead of purging it from the genome, as happens in most invertebrates [48, 49]. As the phylogenetic methods matured and transitioned from parsimony and neighbor-joining to maximum-likelihood and Bayesian analysis methods, so did the methods for compiling TE inventories, which in turn have expanded from dozens to hundreds of thousands of sequences.

If nucleotide or amino acid sequences can be aligned to form reasonably-sized blocks of homology, conventional phylogenetic methods can be applied towards inference of their evolutionary histories. Reconstruction of RT phylogenies began with identification of four and subsequently seven conserved motifs comprising the core domain of RTs and RdRPs, two of which encompass the D,DD catalytic triad [50–53]. These early studies, employing the neighbor-joining and UPGMA methods of tree reconstruction and the Dayhoff distance matrix, already noted the derived nature of most reverse-transcribing viruses and the close relationship between non-LTR retrotransposons and bacterial/organellar group II introns. However, even with the introduction of more advanced phylogenetic analysis methods, such as maximum likelihood and Bayesian analysis, the confidence in resolving deep branches remained far from sufficient, especially when the slower-evolving host genes were combined with the rapidly-evolving sequences of viral origin. For this reason, inclusion of RdRPs in alignments together with host telomerase RTs (TERT) could not yield a definitive answer as to the origin of TERT genes [54, 55]. Nevertheless, inclusion of Penelope-like elements (PLEs) into the RT dataset helped to establish that PLE and TERTs shared a most recent common ancestor when compared with other RTs [56], a finding confirmed by different authors [57, 58].

Conventional phylogenies work reasonably well within and between TE families and superfamilies, and also at higher levels for those TE types which are more prone to vertical transmission and form well-defined clades, such as eukaryotic non-LTR retrotransposons [59]. For these, a semi-automated classification tool based on the BioNJ algorithm, called RTclass1, is available through the web server in Repbase or as a stand-alone tool, and can quickly assign new non-LTR elements to a known clade [60]. For other TE types and for diverse datasets, the assignments can be more complicated. In an ideal world, all TEs should be categorized according to the degree of similarity between extant TE categories and the ancestral forms which gave rise to the more recent branches on the TE evolutionary tree. However, the resolving power of single-gene phylogenies is often insufficient even in the best-case scenario, i.e. assuming uniform rates and the absence of reticulate evolution. Nevertheless, traditional phylogenetic analysis, especially when supplemented with other approaches, can yield some insights into this seemingly unresolvable problem, as evidenced by numerous publications on this topic.

#### Remote homologies

What if the sequences are too distant - can a meaningful analysis still be performed? Does the alignment contain enough phylogenetically informative characters and taxa to prevent artefactual long-branch attraction? Any sequence dataset that is fed into one of the commonly used sequence alignment programs (ClustalW, MUSCLE, MAFFT or T-Coffee [61–64]) is destined to yield an aligned output, even if it consists of largely unrelated sequences. Consequently, if such an alignment is fed into a tree-building program, it will generate a tree with branches and nodes, some of which may occasionally display acceptable branch support values. However, the relevance of such tree-building exercises becomes increasingly doubtful with the decrease in the number of phylogenetically informative characters. It has therefore been argued that attempts to build traditional character-based phylogenetic trees, e.g. for diverse bacterial RTs, are futile, and that the degree of their diversity can only be measured in terms of pairwise distances [65]. Indeed, multiple unidentified and highly diverse RT lineages exist in bacteria, in addition to well-established groups such as retrons, group II introns, related CRISPR/Cas-associated RTs, diversity-generating retroelements (DGR), and Abi (abortive bacteriophage infection)-like genes [65–67]. Some of the unknown groups were assigned to the known ones in an expanded bacterial dataset, leaving 11 unaffiliated lineages [68]. Notably, only group II introns show evidence of autonomous retromobility, while all other RTs are thought to be immobile. Relationship between most bacterial RT lineages remains obscure.

Not surprisingly, RTs were employed as a case study of proteins from what was aptly named the “twilight zone” of sequence similarity with the level of aa identity falling below 20% [58]. In this study, profile-to-sequence comparisons with rps-BLAST yielded an Euclidean distance matrix with resolution of several deep branches that was independent of multiple sequence alignment, but displayed good agreement with alignment-based methods. A similar approach comparing PSI-BLAST scores was used to argue that RNA-dependent RNA polymerases (RdRPs) of eukaryotic positive-strand RNA viruses represent evolutionary descendants of bacterial group II introns, rather than RNA bacteriophages [69, 70]. The exceptionally high evolutionary rates of viral RdRPs, however, complicate elucidation of evolutionary relationships even between RNA viruses themselves, which in addition to RdRP sequences necessitates inclusion of extra non-sequence characters such as specific gene/domain arrangements and the presence/absence of hallmark genes [70].

The problem of character insufficiency is particularly acute for shorter DNA TPases, when compared to RTs: the modest size of DDE-type enzymes and the large degree of flexibility in the spacing of the catalytic D/E residues results in poor resolution of most TPase phylogenies. In an attempt to circumvent the problem, an approach combining the conserved aa “signature string” motifs with additional features, such as target-site duplication (TSD) and terminal inverted repeat (TIR) length/composition, into a binary character matrix has been applied to infer the evolutionary history of the DDE “megafamily” TPases [71]. This approach resulted in merging of some of the original superfamilies into more inclusive ones (e.g. CACTA, Mirage and Chapaev (CMC); PIF/Harbinger and ISL2EU). Evaluation of taxonomic distribution for each superfamily supported the view that the origin of most superfamilies predates the divergence of eukaryotic supergroups.

#### Structure-based alignments and phylogenies

It has long been known that the prior knowledge of the 2-D protein structure can greatly improve the quality of the corresponding alignment and the resulting phylogenetic inferences. Not only can it help to prevent misalignments by avoiding the introduction of improper gaps, which could break apart the conserved secondary structure elements such as α-helices and β-sheets, it can also provide additional information about the degree of similarity for TE-associated proteins, especially for those which lack conserved catalytic residues and are not readily amenable to conventional phylogenetic analysis, e.g. nucleocapsids [72]. Analysis of the most conserved enzymatic components, such as TPases and RTs, can also benefit greatly from structure-based alignments. Below we summarize the current overview for both types, first in the context of between-superfamily relationships and then in comparison with other members of the same protein fold. As a side note, different protein families are grouped into “superfamilies” and then “folds” in the SCOP classification [73], but hereafter the term “superfamily” is used to denote transposon superfamilies, rather than the much broader protein superfamilies and folds.

#### Relationships between DDE transposases

**Fig. 1:**
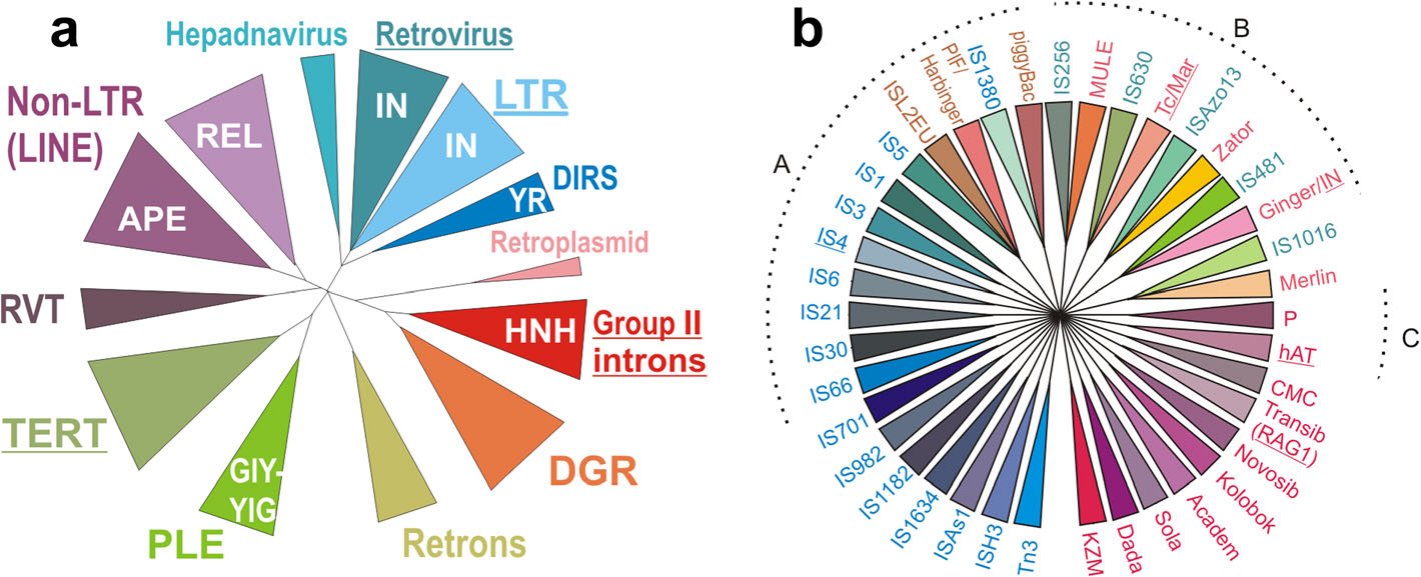
The diversity of reverse transcriptases and DDE transposases found in mobile genetic elements. Groups having representatives with solved 3-D structure are underlined. **a** Phylogenetic analysis of known RTase types (after [88]). In addition to TEs, host genes (TERT, RVT) and non-mobile bacterial RTs are included into the analysis. Also shown are the types of endonucleases/phosphotransferases associated with each RT type. **b** Dendrogram representation of 19 DDE TPase eukaryotic superfamilies from Repbase (www.girinst.org) and 21 prokaryotic DDE families from ISfinder (www-is.biotoul.fr) databases [29, 133] as of this writing. Left, prokaryotic; right, eukaryotic; middle, with cross-domain representation. The dendrogram is star-like, except for cross-domain families with prokaryotic and eukaryotic branches [71, 74, 75]. Bacterial families are in blue/green; eukaryotic in orange/red/purple. Dotted lines denote clades A, B, C from [76]; smaller clades are not shown; assignment of many TEs to known families could not be performed due to the dearth of known representatives. MuA from phage Mu was assigned to clade A, although it is not represented in ISfinder. The more distantly related RuvC-like DEDD TPases of the RNase H family are not included; neither are the mechanistically different HUH, S, Y, or HEN families

For DNA TEs, the best-understood are the TPases from the DDE “megafamily”, named after the conserved Asp-Asp-Glu catalytic triad, which functions to coordinate two divalent metal ions. Other members include retroviral and LTR-retrotransposon integrases (IN), and all of them belong to the larger class of enzymes with an RNase H-like structural fold (which, incidentally, also includes RTs). Hickman et al. [24] performed a comprehensive structure-based comparison of the known DDE TPase superfamilies, integrating prokaryotic and eukaryotic members. The conserved core of the catalytic domain is a mixed alpha-beta fold (β1-β2-β3-α1-β4-α2/3-β5-α4-α5), which beyond the catalytic triad displays negligible sequence similarity between superfamilies, and is also characterized by additional insertions in selected superfamilies. Notably, at least six eukaryotic DDE superfamilies can be paired with related prokaryotic counterparts: Tc/mariner with IS630-like; Merlin with IS1016-like; PIF/Harbinger/ISL2EU with IS5-like; MULE with IS256-like; piggyBac with IS1380-like; and Zator with ISAzo13-like [74, 75] (Fig. 1b). The RNase H-like fold for the superfamilies which were not yet subjected to high-resolution 3-D structural analysis was inferred from secondary structure predictions, with the requirement that the DD of the DDE/D motif falls on or very close to predicted β1 and β4, and the E/D must be on or close to a predicted downstream α-helix. Except for P-element TPases, the presence of RNase H-like fold was confirmed for each superfamily.

#### DDE transposases and the RNase H fold

A broader picture of evolutionary relationships between all groups of RNase H-like enzymes, encompassing not only DDE TPases (including P-elements and RAG genes) and retrovirus-like integrases, but also type 1 and type 2 RNases H, Holliday junction resolvases (including RuvC and CRISPR-associated Cns1 and Cas5e), Piwi/Argonaute nucleases, phage terminases, RNase H domains of Prp8, and various 3′-5′ exonucleases, was presented by Majorek et al. [76]. After initial clustering by pairwise BLAST scores with CLANS [77] and retrieval of additional sequences in profile-HMM searches by HHpred [78], representative multiple sequence alignments were constructed manually, based on the relative positions of the catalytic amino acids and the secondary structure elements. For phylogenetic reconstruction, as expected, the sequence data alone (in which 26 positions showed >40% similarity) could not yield a well-resolved tree, especially given the intermix of prokaryotic and eukaryotic TPases, and had to be supplemented by family similarity scores and catalytic core conservation scores as binary characters in a combined weighted matrix for Bayesian analysis. In this way, RNH-like enzymes were grouped into 12 clades (of which 4 are formed mostly by TPases), with early separation between exo- and endonucleases, as manifested in orientation reversal of the C-terminal α-helix. However, its exclusion from the analysis leads to decrease in resolution within clades; ideally, the subset of endonucleases, with a reference representative added from each known superfamily, as opposed to two randomly selected members, should be re-analyzed using the entire DDE domain to obtain a better picture. High-resolution structures have been obtained only for five types of DDE TPases - Tn5, MuA, Tc/mariner-like (Mos1, Sleeping Beauty, and domesticated SETMAR), Hermes, and retroviral integrases, as well as for RAG recombinase [79–83]. At present, DDE TPase diversity can be depicted only schematically, awaiting availability of additional structural data (Fig. 1b). For other, less representative TPase subclasses, the picture is even more sketchy [38, 84–86].

#### Relationships between reverse transcriptases

In addition to the major prokaryotic RT groups listed above, the following main types of eukaryotic RTs are also distinguished: LTR-retrotransposons and retroviruses; pararetroviruses (hepadna- and caulimoviruses); non-LTR retrotransposons; Penelope-like elements (PLEs); telomerases (TERT); and RVT genes (Fig. 1a). In retroelements, use of structure-based alignments validated by PROMALS3D [87] reinforced the shared ancestry between TERTs and PLEs [88], as well as solidified the common origin of diverse LTR-containing retrotransposons, which in turn have given rise to viruses (retro- and pararetroviruses) at least three times in evolution. The latter ability was associated with acquisition of the RNase H domain by RT, which permits synthesis of dsDNA outside of the nucleus [89]. Also of note are the domesticated RVT genes, which form a very long branch on the RT tree, and harbor a big insertion loop 2a between RT motifs 2 and 3. Their origin remains obscure; notably, this is the only RT group with trans-domain representation, i.e. bacteria and eukaryotes [88].

#### Reverse transcriptases and other right-hand enzymes

In the broader context of right-hand-shaped polymerases (with the characteristic β1-α1-β2-β3-α2-β4 fold of the palm domain), to which RTs belong, the alignment-based phylogenetic matrices are no longer useful, even if supplemented with non-sequence characters. Thus, comparisons are necessarily limited to structure-based distances in a set of proteins with solved high-resolution 3-D structures. A normalized matrix of pairwise evolutionary distances can be obtained using weighted similarity scores, and converted into a tree-like representation. Rather than being limited to a single metric, such as geometric distances (RMSD of the Cα atomic coordinates) or DALI Z-scores (roughly analogous to E-values in BLAST), the combined scores can also incorporate physico-chemical properties of invariant and variable residues in structurally equivalent positions of the structural core, as implemented in the HSF (Homologous Structure Finder) tool [90]. For all right-hand polymerases (RT, viral RdRP, A-, B-, and Y-family DNA polymerases, and T7-like single-subunit RNA polymerases), the common structural core covers 57 α-carbons [91], sharing a common core of 36 residues with more distant superfamilies with a related fold, such as nucleotide cyclases, Prim-Pol, origin-of-replication binding domain, and HUH endonucleases/transposases [92]. In the latter comparison, the processive RNA-dependent (RTs and their sister clade, RdRPs) and DNA-dependent (A-, B-, T7-like) polymerases show distinct separation from the Y-family repair polymerases, which are grouped with nucleotide cyclases. Another study used a non-automated approach to produce a matrix of 26 binary characters to supplement sequence data in right-hand polymerases with known 3-D structure, and yielded similar results except for position of T7-like DNApol; however it included only two RTs (HIV and Mo-MuLV) [93]. Since RNA-dependent polymerization is at the core of the RNA world hypothesis and the transition from RNA- to DNA-based life forms [94], structural investigations of multiple diverse RTs, as opposed to a few select RT structures currently solved, may hold the key to the evolution of early cellular life.

### Domain combinatorics and network analysis

A plausible way to increase phylogenetic resolution within a set of TEs coding for a multi-domain polyprotein would be to perform a combined analysis of all encoded domains. In this way, the phylogenetic signal from the RT can be supplemented with that from PR, RH and IN for LTR retrotransposons, or with EN for non-LTR retrotransposons, yielding higher branch support values [95–97]. However, this approach assumes shared evolutionary history of all polyprotein domains, and therefore each domain should also be evaluated individually for phylogenetic congruence, to avoid superposition of conflicting signals from domains with discordant phylogenies. While the most successful domain combinations can persist throughout long periods of evolution if they confer replicative advantages to a specific group of TEs (e.g. RH-IN in gypsy-like LTR retrotransposons, or AP-endonuclease in non-LTR retrotransposons), non-orthologous domain displacement could yield a convergent evolutionary outcome. As an example, one may consider the RT-RH domain fusion, which endows LTR-retroelements with the ability to escape the confines of the nucleus for completion of dsDNA synthesis in the cytoplasm. RNase H, an enzyme normally available only in the nucleus, has been associated with LTR retrotransposons, retroviruses, and pararetroviruses throughout their evolutionary history, and retroviruses have acquired it twice [89]. Independent acquisitions of an additional RH domain of the archaeal type by LTR and non-LTR retrotransposons have been described recently [98–101], with LTR elements displaying a trend to repeatedly acquire a second RH.

Even within the RT moiety, there may be conflicting views on whether the core RT (fingers and palm) and the thumb domain have always been joined together: despite representing a helical bundle, the thumb domain of telomerases (TERT) markedly differs in structural organization from that of HIV-RT, although they share similar functions [102]. Indeed, the substrate-bound catalytic core of a group II intron LtrA is more similar to that of TERT, while its thumb domain is more similar to that of Prp8, which is responsible for interaction with U5 snRNA [41, 103]. The core RT domain of three other G2Is (including N-terminus) showed similarity to viral RdRPs [104, 105]. While these discrepancies may indicate modular evolution and/or different selective pressures causing structural changes (i.e. non-catalytic nature of Prp8 core), only a comprehensive 3-D structural picture of other known RT types (retrons, DGR, LINE, copia/Ty1, HBV, PLE, RVT) may help to resolve their evolutionary relationships. Signs of reticulate evolution are visible in phylogenetic network analysis of the known RTs, including prokaryotic and eukaryotic representatives [88], and might be indicative of domain swapping.

For complex TEs encoding multiple ORFs, this concern would be even more pronounced, with similar ORFs either co-evolving with others, or being lost and replaced. In recently described giant Terminon retroelements of rotifers, the GIY-YIG-like and structural CC-ORFs appear to evolve concordantly with RTs, while the Rep-like ORFs show discordant evolutionary patterns, indicative of transient association [16]. In DNA-based Polintons, the cysteine protease, ATPase and two major structural proteins, along with pPolB and IN, represent the core components, while other proteins are optional; together, they form part of an extended gene network which also includes virophages, adenoviruses, mitochondrial and cytoplasmic linear plasmids, and Megavirales [106]. Overall, reticulated evolution is frequently observed in TE-encoded ORFs, resulting in network-like patterns rather than bifurcating trees.

### The TE-virus interface

An important dimension which connects TEs with the viral universe is provided by the acquisition of genes which are responsible for nucleoprotein particle formation and interaction with the host cell surface, permitting entry and egress. For RNA-based class I TEs, this dimension is provided by envelope (*env*) genes, which are responsible for interaction with host cell membranes. Their capture by LTR-retrotransposons has occurred independently multiple times in evolution, with the most prominent branch represented by vertebrate retroviruses, supplemented by an impressive diversity of smaller branches in insects, nematodes, and rotifers, with *env* genes acquired from baculoviruses (dsDNA), herpesviruses (dsDNA), phleboviruses (ssRNA), or paramyxoviruses (−ssRNA) [107, 108]. It should be noted that while *env* genes in LTR retrotransposons appear downstream of *pol* as ORF3, acquisition of a downstream ORF3 does not automatically imply that it codes for an *env* gene. The *env*-like function of ORF3’s in numerous plant LTR retrotransposons still has not been established, and in rotifers ORF3s were derived from other enzymatic functions, such as DEDDy exonuclease or GDSL esterase/lipase [108–110]. The nucleocapsid ORFs constitute another important component in retroelement replication, whether they proliferate as enveloped viruses, or intragenomically as ribonucleoprotein particles (RNP), which can form nucleoprotein cores and adopt the shape of virus-like particles (VLPs). The nucleocapsids of retroviruses, caulimoviruses, gypsy-like LTR retrotransposons, and copia-like LTR retrotransposons are thought to be homologous [111], while in other viruses capsid proteins have been evolving many times independently from various host-encoded proteins, including degenerated enzymes [112, 113].

For DNA-based class II TEs, the viral connection is best exemplified by Polintons/ Mavericks, which carry a protein-primed DNA polymerase of the B-family (pPolB) as the replicative component, and a retrovirus/retrotransposon-like integrase (IN, or RVE) as the integrative component [22, 114, 115]. These large TEs, 15–20 kb in length, with terminal inverted repeats, can harbor up to 10 genes, including a cysteine protease and a genome-packaging ATPase with homologs in dsDNA viruses. They occur throughout the eukaryotic kingdom, from protists to vertebrates, and are particularly abundant in the parabasalid *Trichomonas vaginalis*, where they occupy nearly one-third of the genome [115]. While their structural relatedness to DNA viruses, such as adenoviruses, and to cytoplasmic/mitochondrial linear plasmids has been noted early on, the relationship was cemented with detection of a Polinton-like virophage, *Mavirus*, in the flagellate *Cafeteria roenbergensis* [116]. Indeed, homology to the major and minor jelly-roll capsid proteins was detected in Polintons by profile-HMM searches, prompting their designation as Polintoviruses [117]. Nevertheless, these mobile elements are very ancient and constitute an integral part of many eukaryotic genomes, with the principal enzymatic components (pPolB and RVE) evolving congruently and forming deep-branching lineages [118].

Another superfamily of self-replicating TEs, casposons, was recently described in archaeal and bacterial genomes [119]. In addition to pPolB, which represents the replicative component, these elements code for a Cas1 endonuclease, which is also a key component of the prokaryotic CRISPR/Cas adaptive immunity system. Indeed, the casposon-associated Cas1 (casposase) was shown to be functional as a DNA integrase in vitro and to recognize TIRs [120]. In the broader evolutionary picture of self-replicating TEs based on pPolB phylogenetic analysis, pPolB’s from casposons are grouped with archaeal and bacterial viruses, while Polintons may have evolved at the onset of eukaryogenesis, and may have given rise to cytoplasmic linear plasmids and to several families of eukaryotic DNA viruses, including virophages, adenoviruses, and Megavirales [106]. Acquisition of the RVE integrase, however, was apparently the key event in shifting the balance towards intragenomic proliferation of Polintons, and successful colonization of eukaryotic genomes by these TEs.

Most recently, adoption of the TE lifestyle by herpesviruses through co-option of the piggyBac DDE TPase was reported in fish genomes [121, 122]. In this way, a huge (180-kb) viral genome, framed by TIRs recognized by the internally located pBac TPase, became capable of integrating into the genome and causing insertional mutations. Again, combination of the replicative and structural components of a herpesvirus with the integrative component of a DNA TE led to the emergence and proliferation of a new mobile genomic constituent, which may eventually lose its virus-like properties. This process can be regarded as virus domestication [123]. Recruitment of various TPases by viruses has repeatedly occurred in bacteria, resulting in acquisition of the ability to integrate into chromosomes [124].

### An overview of the proposed TE classification as a three-component system

Based on the overview of the existing TE classification systems and the findings summarized above, it would be appropriate and timely to consider TE classification which is based on the ***three element-encoded functions most germane to its proliferative capacity: replicative, integrative, and structural***, the latter also being responsible for intra- or intercellular trafficking. The first two are enzymatic in nature, while the latter are largely non-enzymatic, and thus exhibit more conservation in structure rather than sequence. In addition to these components, TEs may encode other enzymatic or structural functions which may affect the efficiency of TE proliferation and/or the degree of host suppression. Furthermore, TEs may carry passenger genes that may be of use to the host (e.g. antibiotic resistance genes or toxins), or any other cargo genes which happened to be internalized within the transposing unit. None of these, however, are critical for the core mobility functions, and are therefore much less relevant for classification purposes, since they can appear and disappear sporadically.

**Fig. 2:**
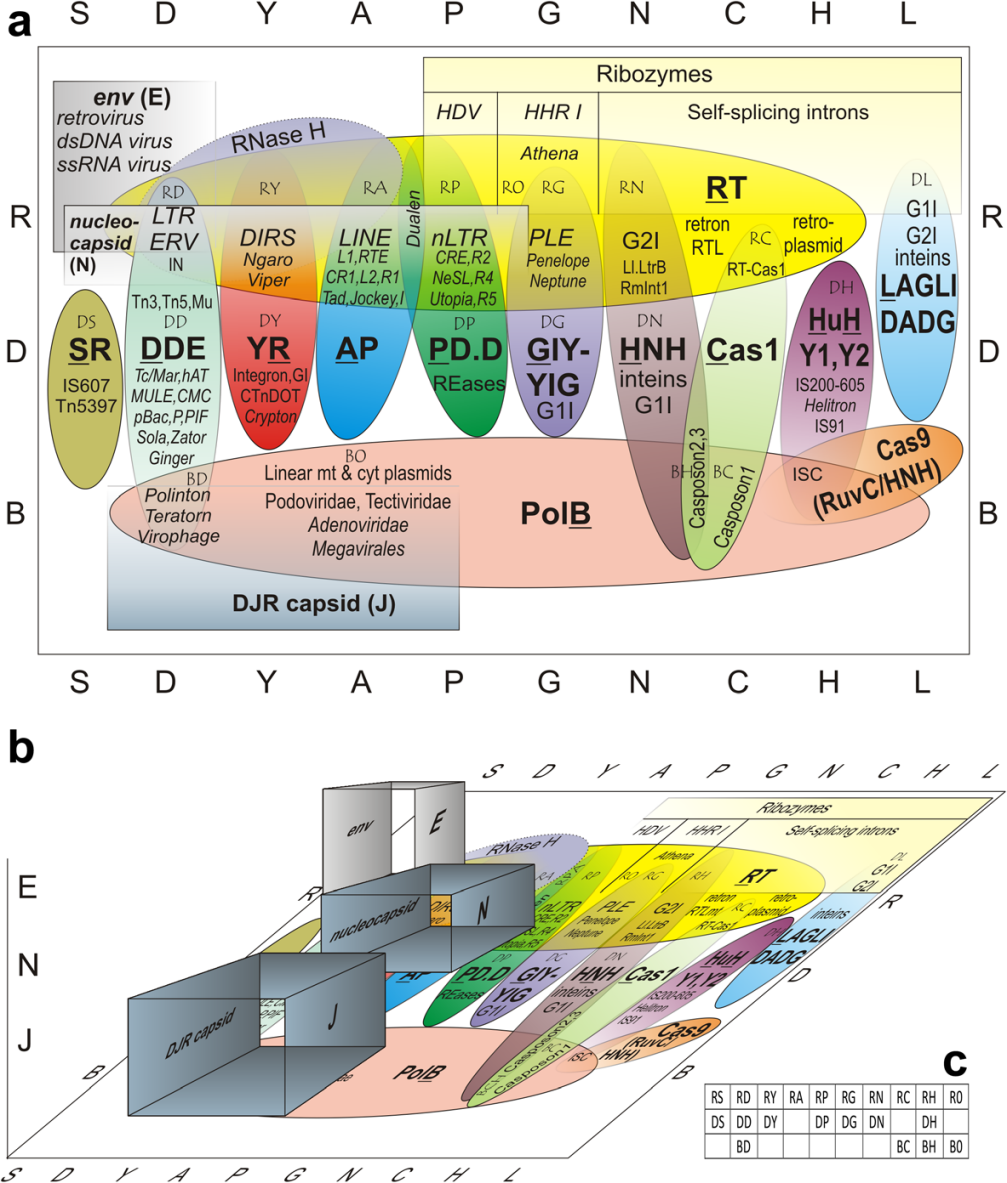
Graphical representation of the replicative, integrative, and structural components contributing to TE diversity. **a** Diversity of polymerase-phosphotransferase combinations in mobile elements. The main types of polymerases and endonucleases are in boldface, and are also shown in single-letter codes along the two respective axes. Two-letter combinations are shown for each TE type at the intersections. **b** Same, with addition of structural components in the third dimension. **c** A 2-D grid listing the currently known combinations of polymerases and endonucleases. A few additional types of endonucleases found only in group I introns are not shown for simplicity

Fig. 2a projects the diversity of TEs, both prokaryotic and eukaryotic, on a two-dimensional grid. The lettered columns correspond to various integrative components, i.e. nucleases/phosphotransferases (or their RNA equivalents with ribozyme activity), and the rows (R, B, or D) correspond to the polymerizing components; for DNA TEs lacking any polymerases and carrying the integrative components only, a D in the first position is preserved. The overlap of Pol and Int types, i.e. replicators and integrators, or lack thereof, creates a distinct TE category at each intersection. Their occurrence on the 2-D grid is symbolized by intersecting ovals, whereas the square-shaped structural components representing capsid and envelope proteins (E, N, J) may be extended into the third dimension, as they can potentially give rise to virus-like entities, and/or facilitate intra- and intercellular movements (Fig. 2b). Note that the scheme can be expanded in any of the directions to accommodate additional types of polymerases and integrases, as well as any novel types of structural components. It also helps to alleviate the duality of assignment caused by the presence of different polymerase and integrase types in a single element. It would be of interest to find out whether any previously undescribed combinations can in fact be discovered in the vast diversity of sequenced life forms, may evolve over evolutionary time, or exist in the form of molecular fossils.

In practice, consideration may be given by the community of TE annotators to adjusting the three-letter code [11], which is already used by some programs, but rarely utilizes all three positions. If the type of polymerase is denoted by the first letter, and the type of endonuclease/phosphotransferase by the second letter (Fig. 2c), with D in the first position denoting the lack of the polymerizing component, and O reserved for the absence of integrating component (as in EN(−) telomere-attaching retroelements [125] or a subset of group II introns [68]), it may endow the current code with additional biological meaning. The type of structural protein might be designated by the third letter, however the problem of recognition of rapidly evolving structural components that do not exhibit much sequence conservation diminishes its practical value. Nevertheless, there are still possibilities to include subclasses/superfamilies in the code, and/or accommodate any ribozyme components. Regardless of practical outcomes, it is useful to consider each of the three aspects of TE proliferation as a different dimension. As for the concern expressed in [6] that viruses should not be regarded as TEs if they can serve as vectors to transfer other TEs, in this way a substantial part of the mobilome could be eliminated. Overall, any DNA that can propagate in the genome without an obligatory external stage should be regarded as a component of the mobilome.

### Concluding remarks

In the past decade, we have witnessed a major transition in the process of discovery of new types of TEs. Originally, it was driven by experimental observations, whereby TE mobility was associated with certain phenotypic changes. At present, bioinformatic investigations became front and center of TE discovery, opening the window into identification and characterization of giant transposable units, broadly categorized as genomic islands, which have previously escaped detection, and shifting the balance of forces thought to play major roles in shaping and re-shaping ancient and modern genomes. TPases and RTs are arguably the most abundant genes on Earth, depending on the counting method [126, 127], and novel TE superfamilies, such as Zisupton/KDZ, continue to be discovered [128, 129]. Experimental validations and applications of bioinformatic findings in vivo and in vitro are somewhat lagging, and more resources need to be invested in biological experimentation to achieve better understanding of genome-mobilome interactions and their consequences.

An important experimental area in which progress should be encouraged is the generation of a comprehensive structural picture in which a representative of each major TE superfamily (subclass) is associated with a high-resolution 3-D structure. In the age of the cryo-EM revolution [130], such an initiative, which can be thought of as the “Structural 3-D challenge” for TEs, would certainly be justified, and could eventually result in generating a “tree of life” for both DNA and RNA TEs, by analogy with the organismal Tree of Life initiative. Another area which may shed light on the mobilome function is the advance of synthetic genomics, which may allow construction of entirely repeat-free artificial genomes, giving rise to host species free of any TEs. It would be of much interest to evaluate their adaptive potential, and to find out for how long would such species be able to stay TE-free.

Many outstanding questions remain to be explored bioinformatically. For example, a comprehensive database of profile HMMs for each TE family at the protein level has not been compiled. The Dfam database of repetitive DNA families includes DNA profile HMMs for five model species (human, mouse, zebrafish, fruit fly and nematode) [131]. However, the amino acid profile HMMs constitute parts of the larger protein databases such as Pfam or CDD, where they are not always explicitly designated as TEs. Development of de novo TE identification tools should be accompanied by a coordinated effort in benchmarking TE annotation methods [132]. Expansion of metagenomic datasets may help to answer interesting questions such as whether each eukaryotic DNA TE superfamily can be matched with a prokaryotic counterpart, and how may RT and polymerase types can give rise to viruses. Finally, modification of the current one-dimensional TE classification system into a broader one accommodating replication, integration/excision, and intra/intercellular mobility dimensions of the TE life cycle may be regarded as the “Classification 3-D challenge”. Overcoming these challenges could raise the science of comparative genomics to a new level, and bring us closer to understanding the full impact of TEs on genome structure, function, and evolution.

## Abbreviations

Aa: amino acid
AP: Apurinic-Apyrimidinic endonuclease
CDD: Conserved Domain Database
DGR: Diversity-Generating Retroelements
EN: Endonuclease
ERV: Endogenous Retrovirus
G2I: Group II Introns
HEN: Homing Endonuclease
HMM: Hidden Markov Model
IN: Integrase
LINE: Long Interspersed Element
LTR: Long Terminal Repeat
MGE: Mobile Genetic Element
PLE: Penelope-Like Element
PR: Protease
RCR: Rolling-Circle Replication
RdRP: RNA-dependent RNA polymerase
REL: Restriction Enzyme-Like endonuclease
RH: RNase H
RMSD: Root Mean Square Deviation
RNP: Ribonucleoprotein Particle
RT: Reverse Transcriptase
SCOP: Structural Classification of Proteins
TE: Transposable Element
TERT: Telomerase Reverse Transcriptase
TIR: Terminal Inverted Repeat
TPase: Transposase
TPRT: Target-primed Reverse Transcription
TSD: Target Site Duplication
VLP: Virus-Like Particles
YR: Tyrosine Recombinase
